# The effect of ultrasound-guided low serratus anterior plane block on laparoscopic cholecystectomy postoperative analgesia: A randomized clinical trial

**DOI:** 10.1097/MD.0000000000027708

**Published:** 2021-11-05

**Authors:** Yu Wu, Weicai Yang, Zenghua Cai, Zaiwang Zhang

**Affiliations:** a Department of Anesthesiology, No. 980th Hospital (Bethune International Peace Hospital) of Joint Logistic Support Force, Shijiazhuang, China; b Department of Anesthesiology, First People's Hospital of Xiangtan City, Xiangtan, China.

**Keywords:** laparoscopic cholecystectomy, postoperative analgesia, serratus anterior plane block

## Abstract

**Background:**

Anterior serratus plane block for analgesia in many procedures, but there have been no reports of analgesia undergoing laparoscopic cholecystectomy (LC). We investigated the effects of ultrasonic-guided low serratory anterior block on patients undergoing LC.

**Methods:**

One hundred patients who undergo LC were selected and randomly divided into 2 groups: Patients in group C with routine general anesthesia and patients in group S treat low anterior serratus block combined with general anesthesia. The serratus anterior block was performed at the T8 to T9 point after anesthesia induction, before cutting leather (T0), stamp card placement (T1), 2 groups of patients’ heart rate (HR), blood pressure were recorded, at the same time dosage of analgesic drugs and postoperative 0.5, 6, 12, 24 hours when resting visual analogue scale (VAS) scores were recorded in 2 groups of patients.

**Results:**

The T0 point, patients’ HR, blood pressure had significant difference (*P* > .05), the T1 point, patients’ HR, mean arterial pressure in group S lower than group C (*P* < .05), the amount of intra-operative propofol and remifentanil, patients in group S were less than in group C (*P* < .05), and resting VAS pain score at the 0.5, 6, 12 hours after operation patients in group S were lower than group C also (*P* < .05), resting VAS pain had no significant difference at postoperative 24 hours between 2 groups (*P* > .05).

**Conclusion:**

Low serratus anterior plane block in LC can provide safe and effective analgesia for patients.

## 1 Introduction

Compared with traditional open surgery, laparoscopy has the characteristics of small trauma, clear field of vision, explicit operation and rapid postoperative recovery, etc, which can perfectly meet the requirements of enhanced recovery after surgery.^[1]^ Therefore, it has developed rapidly in recent years and has become the mainstream means of clinical surgical operation. Laparoscopic cholecystectomy (LC) has been used for many years and has completely replaced the traditional open cholecystectomy. Although the degree of trauma and postoperative pain in LC is far lower than that in open surgery, pneumoperitoneum and puncture card implantation can still cause postoperative pain in patients.^[2]^

Postoperative pain is a reaction of the body to injury stimulation such as surgery. The occurrence of postoperative pain leads to a series of pathological and physiological changes, aggravating the psychological burden of patients. Serious pain even leads to severe adverse reactions such as depression, which is contrary to the comfort medical treatment common pursuit of doctors and patients now. Pain can show accelerated heart rate (HR) and elevated blood pressure, and even cause serious adverse outcomes of heart failure and myocardial infarction.^[3,4]^

Peripheral nerve block can effectively relieve acute postoperative pain and reduce the intra-operative use of opioids in patients with LC. Serratus anterior plane block (SAPB) is a kind of peripheral nerve block, which is considered to be a safe, effective, easy to operate analgesic method with low risk of side effects.^[3,5]^ Therefore, this study investigated the effects of SAPB on intra-operative hemodynamics and pain relief in undergoing LC patients. Pain is a subjective experience, which can be influenced by physiological, psychological, personal experience, social and cultural factors. The perception of, expression of, and reaction to pain are influenced by genetic, developmental, familial, psychological, social and cultural variables.^[6]^ The visual analogue scale (VAS) score is most commonly used for pain assessment,^[7]^ then the primary endpoint of this research was VAS scores and secondary endpoint were dosage of propofol or remifentanil and variation in blood pressure or HR in patients.

## 2 Materials and methods

This study was approved by the Ethics Committee of No. 980 Hospital of the Joint Logistic Support Force of the People's Liberation Army of China and The First People's Hospital of Xiangtan City. Inclusion criteria: Older than 18 years; The patient agreed and signed the informed consent. Exclusion criteria: History of allergy to anesthetic drugs; Cardiac function grade III or IV; Liver function changes (Child-Pugh grade B or above); The body temperature is higher than 38°C or lower than 36°C; Blood routine showed greater than 4 × 10^9^ or less than 10 × 10^9^ leukocytes.

Patients were randomly divided into conventional general anesthesia group (group C) and low serratus anterior block combined with general anesthesia group (group S) by random envelope method, neither the patient nor the follow-up physician was aware of the grouping. All patients were given intramuscular injection of diazepam 10 mg and atropine 0.5 mg when they were brought into the operating room. Patients in Group C were treated by routine intravenous general anesthesia, midazolam, 0.04 mg kg^−1^, sufentanil 0.5 μg kg^−1^, propofol 2.5 mg kg^−1^, cisatracurium besilate 0.3 mg kg^−1^. When induced, the laryngeal mask was placed for mechanical ventilation, and then setting: tidal volume: 6 to 8 mL/kg, respiratory rate: 12 to 16 times/min, maintain PetCO_2_ between 35 and 45 mm Hg. The radial artery on the left side was given invasive arterial pressure monitor. Patients in group S were treated with low serratus anterior (T8–T9) plane block on right after anesthesia induction. The patient was supine with the upper arm abducted on right and the probe was placed on the sagittal plane of the midline of the right clavicle of the chest. The ribs were counted from the lower side, and the cloth was disinfected and laid with a portable ultrasound high-frequency linear array probe. When the tip reached between the serraspus anterior and the intercostal muscles, the syringe was extracted without blood and the position of the tip was confirmed. After that, 30 mL of 0.375% ropidocaine injection was given, and the chest wall of the injection site was gently pressed to accelerate the diffusion. During the operation, propofol (4 mg kg^−1^ h^−1^) and remifentanil (0.2 μg kg^−1^ min^−1^) were pumped continuously through intravenous infusion to maintain anesthesia. During the operation, HR and mean arterial pressure (MAP) were recorded before the incision and placement of poking cards (T0) and after the placement of all poking cards (T1) in the 2 groups, and the intra-operative dosage of remifentanil and propofol in the 2 groups were recorded. Blood pressure, HR, peripheral oxygen saturation, end-expiratory partial carbon dioxide pressure, and bispectral index were monitored at the same time. The bispectral index value was maintained at 40 to 50 by adjusting the dose and infusion rate. Postsurgery, patients were given 5 mg dezocine, 5 μg sufentanil, and 5 mg tolisetron, the laryngeal mask was removed successfully and the patients returned safely to the ward.

The VAS scores, the primary outcome, adverse reactions and complications at resting time of 0.5, 6, 12, and 24 hours were followed up by non-participating anesthetic nurses in both groups. If the VAS score was ≥4, 5 mg of dezocine was injected intravenously by the nurses in the ward, and the incidence of complications related to nerve block and puncture (hematoma, infection, etc) within 24 hours after surgery was recorded.

### 2.1 Statistical analysis

SPSS (Version 22.0) statistical package (IBM Corporation, Armonk, NY) was used for statistical analysis. Measurement data were expressed as mean ± standard (x ± s) deviation. Independent-sample *t* test was used for inter-group comparison, and repeated measurement data analysis of variance was used for intra-group comparison. Enumerative data were expressed as the ratio or percentage, χ2 test was used, and the parametric data that did not conform to the normality distribution were represented as the median (quaternary) [M(Q1,Q3)], *P* < .05 was considered statistically significant.

## 3 Results

1. One hundred four patients who planned to LC from October 2019 to February 2021 were selected for this study (80 patients from No. 980 Hospital, 20 patients from First People's Hospital of Xiangtan City), 1 patient was excluded from the study because of switching to open surgery, and 3 patients were excluded for temperature >38.0°C. There was no statistical significance in age, gender, body mass index, American Society of Anesthesiologists classification and operation time between the 2 groups (*P* > .05, Table 1).
2. Hemodynamics before dermectomy (T0) and the puncture card was placed (T1) between the 2 groups: there were no statistically significant differences in HR and MAP between the 2o groups at T0 (*P* > .05), but HR and MAP were increased in group S's patients and group C's patients when compared with T0 (*P* < .05). At T1 point, HR in group S's patients was lower than that in group C's patients (*P* < .05), but there was no statistical significance in MAP (*P* > .05, as shown in Table 2).
3. Comparison of intra-operative amounts of propofol and remifentanil: the amounts of propofol and remifentanil in group S's patients were lower than those in group C's patients (*P* < .05, Table 3).
4. Comparison of VAS at different postoperative time points: compared with group C's patients, VAS at 0.5, 6, and 12 hours postoperative time points in group S's patients was lower (*P* < .05). Only 2 patients in group C's patients were given 5 mg dezocine intramuscular injection at 12 hours postoperative because of resting VAS > 4 points. However, at 24 hours postoperative time points, there was no statistically significant difference in VAS between the 2 groups, and no complications related to puncture site occurred in group S (*P* > .05, Table 4).

**Table 1:** Comparison of characteristics of patients [x ± s].

	Age (yr)	Gender (F/M)	BMI (kg/m^2^)	ASA (n) (I/II/III)	Operation time (min)
Group C (n = 50)	60.06 ± 9.58	22/28	31.39 ± 4.52	10/30/10	54.00 ± 5.97
Group S (n = 50)	61.66 ± 7.78	23/27	30.67 ± 3.83	8/31/11	55.42 ± 5.03
t	−0.65	0.40	0.86	0.29	−1.29
*P*	.52	.84	.39	.87	.20

Values are presented as mean ± SD, categorical variables are presented as numbers and percentages. ASA = American Society of Anesthesiologists, BMI = body mass index, F = female, M = male, n = number, SD = standard deviation.

**Table 2:** Comparison of mean arterial pressure and heart rate (x ± s).

	Mean arterial pressure, mm Hg		Heart rate (/min)	
	T0	T1	T0	T1
Group C (n = 50)	84.08 ± 6.08	95.63 ± 7.56	71.42 ± 5.81	78.52 ± 4.23
Group S (n = 50)	85.46 ± 4.99	92.89 ± 5.49	70.46 ± 5.42	76.57 ± 4.81
t	−1.24	2.08	0.85	2.15
*P*	.22	.04	.40	.03

**Table 3:** Comparison of intra-operative amounts of propofol and remifentanil (x ± s).

	Propofol (mg)	Remifentanil (μg)
Group C (n = 50)	301.55 ± 34.25	795.51 ± 75.27
Group S (n = 50)	300.57 ± 29.17	755.60 ± 90.26
t	2.13	2.40
*P*	.04	.02

**Table 4:** Comparison of VAS at different time points between the 2 groups ([M(Q1,Q3)]).

	0.5 h	6 h	12 h	24 h
Group C (n = 50)	1.44 (1–2)	1.90 (1–2)	1.86 (1–2)	1.40 (1–2)
Group S (n = 50)	1.30 (1–2)	1.56 (1–2)	1.601–2)	1.44 (1–2)
t	1.45	2.94	2.09	−0.39
*P*	.15	.004	.04	.70

VAS = visual analogue scale.

## 4 Discussion

LC has now become a routine operation and is also the preferred method for the treatment of benign gallbladder diseases.^[8]^ In order to speed up hospital bed turnover and reduce the medical and economic burden of patients, many hospitals have begun to try to perform LC in day wards.^[9]^ However, the early pain postcholecystectomy seriously affects the recovery of patients and even leads to patients to be transferred from the day ward to the ward for further treatment,^[10]^ which is also contrary to the concept of accelerated rehabilitation surgery. Therefore, good analgesia after LC is particularly important.

The traditional analgesic method for abdominal surgery is epidural block, but its application is limited due to its high requirements on coagulation function and relatively large risk,^[11]^ and opioids is still the commonly used analgesic method at present. Peripheral nerve block has low requirement to the function of blood coagulation, the advantages of the operation is relatively simple, gradually appeared by ultrasound guiding clinical abdominal transverse plane block (transversus abdominis plane) for LC and postoperative analgesia,^[12,13]^ has obtained the certain effect, but still cannot achieve complete remission patients pain stimulus.

Musculus serratus anterior is located subcutaneously on the surface of lateral thoracic wall, and the upper part is covered by pectoralis major and pectoralis minor. Musculus serratus starts from the 1st to 9th ribs and terminates at the spinal margin of scapula.^[14]^ Saw muscle plane block before the first put forward by Blanco et al,^[15]^ in the 4 women volunteers in axillary midline before under the guidance of ultrasound in the fifth rib saw muscle injection of local anesthetic drug clearance, volunteers block plane finally reach the intercostals nerve T2 to T9, intercostals nerve area up to 752 minutes in a block of time, in the motor nerve block time of 778 minutes. Due to the simple operation of anterior serratory muscle plane block and the low probability of pneumothorax and local anesthetic poisoning, it has been gradually applied in clinical practice^[16]^ and is now widely used in the postoperative analgesia treatment of breast surgery, such as radical breast cancer,^[17]^ breast plastic surgery,^[18]^ and thoracoscopic surgery.^[19]^ Although recent studies^[17]^ have shown that in breast cancer patients, the addition of serratory anterior plane block on the basis of general analgesia cannot improve the quality of patients’ rehabilitation. However, there have also been reports on the effects of SAPB on postoperative analgesia, inflammatory factors and immune function of upper abdominal surgery such as open partial hepatectomy,^[20]^ all of which have shown good effects of SAPB.

Opioids have been used to treat and prevent postoperative pain, but opioid-related adverse events lead to poor prognosis.^[21]^ An increasing number of surgical patients are tolerant to opioids (e.g., patients with chronic pain), making it challenging to provide adequate postoperative analgesia while reducing the risk of overdose or recurrence. For these patients, optimize the perioperative use of opioids is likely to have a significant impact on their recovery,^[22]^ line postoperatively in patients with peripheral nerve block to alleviate pain and inflammation and the mechanism of opioid analgesics, as part of the multimodal analgesia strategy, it may help reduce the demand for opioid drugs.

In this study, low level (T8–T9) serratus anterior muscle plane block was used to observe the clinical effect of intra-operative and postoperative analgesia during LC. Patients’ intra-operative HR and blood pressure can reflect the stress degree of patients to external stimuli, which is also a direct response to the effect and depth of anesthesia. Our results showed that right low level serratory anterior muscle plane block can effectively reduce the stimulation during skin cutting and puncture card placement during LC, maintain a more stable HR and blood pressure, and facilitate the hemodynamic stability of patients during LC. At the same time, SAPB can provide patients with more adequate intra-operative analgesia and reduce the opioids used during the operation, which is conducive to the recovery of intestinal function of patients, more in line with the requirements of accelerated rehabilitation surgery, and conducive to the early recovery of patients.^[23]^ At the same time, our study also found that the intra-operative sedative propofol in patients with serratory anterior plane block also decreased, and patients only needed less sedative and analgesic to achieve a deeper anesthesia depth, meet the anesthesia requirements of patients, and avoid the occurrence of related side effects after the use of a large number of anesthesia drugs. For patients with postoperative follow-up found resting VAS score, 0.5 hours 2 groups of patients with postoperative left the postoperative recovery room did not differ between the VAS score, it may be patients were given a dose of analgesic drugs, analgesic drug in the body is still in the analgesic effect of the blood drug concentration, so there is no difference between the VAS score. And postoperative 6 hours time point, analgesic drugs began to slowly fall to below the effective blood drug concentration, anterior muscle plane block in the conventional VAS score was lower than that patients with only obtained general anesthesia group, anterior muscle plane block in patients with LC has good analgesic effect, and the blocking effect can continue until postoperative 12 hours, which confirmed the Blanco et al^[15]^ reports block time can reach 752 minutes. However, as time goes by, the concentration of ropivacaine enhancement of metabolism, the postoperative VAS scores between the 2 groups gradually converged, and the difference between the 2 groups had no statistical significance at postoperative 24 hours time point. The VAS scores at 24 hours were significantly lower than those at 12 hours, suggesting that we need to control the pain in patients with LC in the early stage.

### 4.1 Limitations

There are still some deficiencies in our study. First of all, the inclusion criteria of our study did not distinguish the primary diseases of the gallbladder. The second, our study did not assess the actual extent of the block in patients undergoing SAPB. Finally, the types and concentrations of local anesthetics used in SAPB are single, and there is a lack of research on different local anesthetics and different concentrations. More research is needed to confirm all of this.

## 5 Conclusion

The SAPB can bring good analgesic effect for patients undergoing LC during and postoperation, without obvious adverse reactions, which is worthy of clinical promotion.

## Author contributions

**Conceptualization:** Yu Wu, Zenghua Cai.

**Data curation:** Yu Wu, Weicai Yang, Zenghua Cai.

**Formal analysis:** Yu Wu, Zenghua Cai.

**Investigation:** Yu Wu, Weicai Yang.

**Methodology:** Yu Wu, Weicai Yang.

**Resources:** Yu Wu, Zenghua Cai.

**Software:** Yu Wu.

**Supervision:** Yu Wu, Zaiwang Zhang.

**Validation:** Yu Wu.

**Visualization:** Yu Wu, Zaiwang Zhang.

**Writing – original draft:** Yu Wu.

**Writing – review & editing:** Yu Wu, Zaiwang Zhang.

## References

[1] Najjar PA Fields AC Maldonado LJ Ward A Bleday R. Differential index-hospitalization cost center impact of enhanced recovery after surgery program implementation. Dis Colon Rectum 2020;63:837–41. 32168094 10.1097/DCR.0000000000001662

[2] Gurusamy KS Vaughan J Toon CD Davidson BR. Pharmacological interventions for prevention or treatment of postoperative pain in people undergoing laparoscopic cholecystectomy. Cochrane Database Syst Rev 2014. CD008261. 24683057 10.1002/14651858.CD008261.pub2 PMC11086628

[3] de Oliveira Filho GR Kammer RS Dos Santos HC. Duloxetine for the treatment acute postoperative pain in adult patients: a systematic review with meta-analysis. J Clin Anesth 2020;63:109785. 32179396 10.1016/j.jclinane.2020.109785

[4] Braun M Bello C Riva T. Quantitative sensory testing to predict postoperative pain. Curr Pain Headache Rep 2021;25:03. 10.1007/s11916-020-00920-5 PMC7808998 33443676

[5] Elsabeeny WY Ibrahim MA Shehab NN Mohamed A Wadod MA. Serratus anterior plane block and erector spinae plane block versus thoracic epidural analgesia for perioperative thoracotomy pain control: a randomized controlled study. J Cardiothorac Vasc Anesth 2021;35:2928–36. 33483269 10.1053/j.jvca.2020.12.047

[6] Raja SN Carr DB Cohen M. The revised International Association for the Study of Pain definition of pain: concepts, challenges, and compromises. Pain 2020;161:1976–82. 32694387 10.1097/j.pain.0000000000001939 PMC7680716

[7] Myles PS Myles DB Galagher W. Measuring acute postoperative pain using the visual analog scale: the minimal clinically important difference and patient acceptable symptom state. Br J Anaesth 2017;118:424–9. 28186223 10.1093/bja/aew466

[8] Cao J Liu B Shi J. Safety of ambulatory laparoscopic cholecystectomy in the elderly. ANZ J Surg 2021;91:597–602. 33605041 10.1111/ans.16656

[9] Umemura A Suto T Nakamura S. Comparison of single-incision laparoscopic cholecystectomy versus needlescopic cholecystectomy: a single institutional randomized clinical trial. Dig Surg 2019;36:53–8. 29393173 10.1159/000486455

[10] Rosero EB Joshi GP. Hospital readmission after ambulatory laparoscopic cholecystectomy: incidence and predictors. J Surg Res 2017;219:108–15. 29078868 10.1016/j.jss.2017.05.071

[11] Qin C Liu Y Xiong J. The analgesic efficacy compared ultrasound-guided continuous transverse abdominis plane block with epidural analgesia following abdominal surgery: a systematic review and meta-analysis of randomized controlled trials. BMC Anesthesiol 2020;20:52. 32111162 10.1186/s12871-020-00969-0 PMC7048149

[12] Karasu D Yilmaz C Ozgunay SE Yalcin D Ozkaya G. Ultrasound-guided transversus abdominis plane block for postoperative analgesia in laparoscopic cholecystectomy: a retrospective study. North Clin Istanb 2021;8:88–94. 33623879 10.14744/nci.2020.84665 PMC7881418

[13] El-Dawlatly AA Turkistani A Kettner SC. Ultrasound-guided transversus abdominis plane block: description of a new technique and comparison with conventional systemic analgesia during laparoscopic cholecystectomy. Br J Anaesth 2009;102:763–7. 19376789 10.1093/bja/aep067

[14] Lung K St Lucia K Lui F. Anatomy, Thorax, Serratus Anterior Muscles. Treasure Island, FL: StatPearls; 2021. 30285352

[15] Blanco R Parras T McDonnell JG Prats-Galino A. Serratus plane block: a novel ultrasound-guided thoracic wall nerve block. Anaesthesia 2013;68:1107–13. 23923989 10.1111/anae.12344

[16] Liu X Song T Xu HY Chen X Yin P Zhang J. The serratus anterior plane block for analgesia after thoracic surgery: a meta-analysis of randomized controlled trails. Medicine (Baltimore) 2020;99:e20286. 32481308 10.1097/MD.0000000000020286 PMC7249988

[17] Abdallah FW Patel V Madjdpour C Cil T Brull R. Quality of recovery scores in deep serratus anterior plane block vs. sham block in ambulatory breast cancer surgery: a randomised controlled trial. Anaesthesia 2021;76:1190–7. 33492696 10.1111/anae.15373

[18] Khemka R Chakraborty A Ahmed R Datta T Agarwal S. Ultrasound-guided serratus anterior plane block in breast reconstruction surgery. A A Case Rep 2016;6:280–2. 26934607 10.1213/XAA.0000000000000297

[19] Gao W Yang XL Hu JC. Continuous serratus anterior plane block improved early pulmonary function after lung cancer surgery. Ann Thorac Surg 2021. 01–8. doi:10.1016/j.athoracsur.2021.02.032. 10.1016/j.athoracsur.2021.02.032 33667460

[20] Tao KM Xu HH Zhu CC Lu ZJ. Serratus anterior plane block catheter for hepatectomy: a method to decrease opioid use perioperatively. J Clin Anesth 2020;61:109682. 31859134 10.1016/j.jclinane.2019.109682

[21] Martinez L Ekman E Nakhla N. Perioperative opioid-sparing strategies: utility of conventional NSAIDs in adults. Clin Ther 2019;41:2612–28. 31733939 10.1016/j.clinthera.2019.10.002

[22] Kumar K Kirksey MA Duong S Wu CL. A Review of opioid-sparing modalities in perioperative pain management: methods to decrease opioid use postoperatively. Anesth Analg 2017;125:1749–60. 29049119 10.1213/ANE.0000000000002497

[23] Rendon JL Hodson T Skoracki RJ Humeidan M Chao AH. Enhanced recovery after surgery protocols decrease outpatient opioid use in patients undergoing abdominally based microsurgical breast reconstruction. Plast Reconstr Surg 2020;145:645–51. 32097300 10.1097/PRS.0000000000006546

